# Deep Learning Driven Drug Discovery: Tackling Severe Acute Respiratory Syndrome Coronavirus 2

**DOI:** 10.3389/fmicb.2021.739684

**Published:** 2021-10-28

**Authors:** Yang Zhang, Taoyu Ye, Hui Xi, Mario Juhas, Junyi Li

**Affiliations:** ^1^College of Science, Harbin Institute of Technology (Shenzhen), Shenzhen, China; ^2^Medical and Molecular Microbiology Unit, Department of Medicine, Faculty of Science and Medicine, University of Fribourg, Fribourg, Switzerland; ^3^School of Computer Science and Technology, Harbin Institute of Technology (Shenzhen), Shenzhen, China

**Keywords:** deep learning, database, drug discovery, antibiotics, antimalarial drug, drug repurposing, SARS-CoV-2

## Abstract

Deep learning significantly accelerates the drug discovery process, and contributes to global efforts to stop the spread of infectious diseases. Besides enhancing the efficiency of screening of antimicrobial compounds against a broad spectrum of pathogens, deep learning has also the potential to efficiently and reliably identify drug candidates against Severe Acute Respiratory Syndrome Coronavirus 2 (SARS-CoV-2). Consequently, deep learning has been successfully used for the identification of a number of potential drugs against SARS-CoV-2, including Atazanavir, Remdesivir, Kaletra, Enalaprilat, Venetoclax, Posaconazole, Daclatasvir, Ombitasvir, Toremifene, Niclosamide, Dexamethasone, Indomethacin, Pralatrexate, Azithromycin, Palmatine, and Sauchinone. This mini-review discusses recent advances and future perspectives of deep learning-based SARS-CoV-2 drug discovery.

## Introduction

**FIGURE 1:**
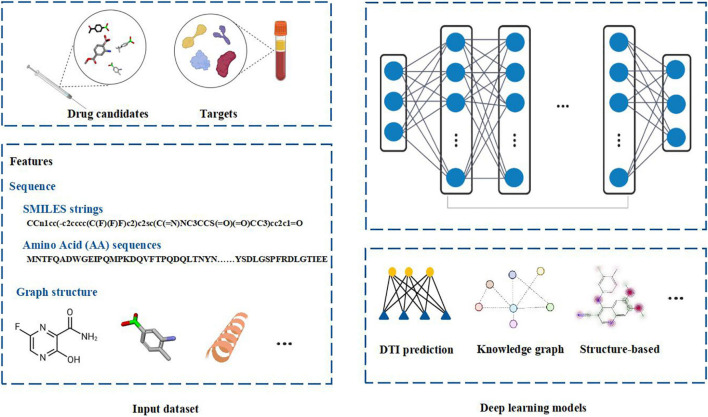
The schematic diagram of deep learning in drug discovery. Biochemical data from drug candidates and protein targets can be used for drug discovery. Chemical sequences (simplified molecular-input line-entry system (SMILES) strings) and amino acid (AA) sequences, structure of chemical compounds and protein targets can be used as the features to fed into the deep learning models. Different deep learning models can be employed to analyze the data by integrating Drug Target Interaction (DTI) prediction, knowledge graph or structure based computational methods. Figure is made in part with BioRender.

Deep learning is a branch of machine learning. It is an algorithm that abstracts data by using multiple processing layers composed of complex structures or multiple non-linear transformations. Compared with the shallow machine learning methods, deep learning algorithm is a process of automatic feature engineering. Deep learning frameworks, such as convolutional neural network and recursive neural network, have been applied in the fields of bioinformatics and biomedicine and achieved excellent results (Lipinski et al., 2019). Deep learning methods have good applications in microbiology including metagenomic data analysis, microbial-related drug discovery, disease microbial association and so on (Duch et al., 2007). When analyzing microbial related data, it shows high prediction accuracy in practice. Because deep learning algorithms are good at obtaining very complex underlying patterns in data, they are especially suitable for large and high-dimensional data sets. Moreover, it is easy to update the model with the new data. The hidden layer of the network obviously reduces the demand for Feature Engineering and is conducive to the completion of prediction tasks. The schematic diagram of the deep learning in drug discovery is shown in Figure 1.

Deep learning has revolutionized most areas of science and technology, including drug discovery. Traditional drug discovery methods are not time and cost efficient and therefore often unable to keep pace with the rapidly emerging and re-emerging pathogenic microorganisms. More recent drug discovery methods include Naive Bayesian, Support Vector Machines and Neural Networks (Bender et al., 2007; Stephenson et al., 2019). These alternative drug discovery methods usually use bigger data sets generated from high throughput screenings and allow more accurate prediction of bioactivities and molecular properties of the targets (Stephenson et al., 2019). Compared to these alternative machine learning methods used for drug discovery, deep learning is characterized by the flexibility of the architecture of Neural Networks (Chen et al., 2018). Given the cost and time required for traditional drug discovery, deep learning has the potential to significantly accelerate the drug discovery process. By using information on the biological, chemical, and topological properties of compounds and their putative targets from the large-scale libraries, deep learning can be employed to identify the most promising drugs against specific diseases (Neves et al., 2020; Stokes et al., 2020). Various deep learning methods have been developed over the last few years, but their application in drug discovery has still not reached its full potential. One of the main hurdles for researchers planning to build their own deep learning model for drug identification is the amount of resources and time required to collect large amounts of data. A number of computational screen open databases have been made to prioritize drug candidates, recently. A representative set of open access datasets which can be used to train deep learning models for specific research projects is shown in Table 1.

**TABLE 1:** Representative biochemical datasets used in deep learning studies.

**Dataset**	**Description**	**URL**	**References**
ZINC	ZINC database contains over 230 million compounds.	http://zinc.docking.org/	Bai et al., 2020; Choi et al., 2020; Stokes et al., 2020; Ton et al., 2020
ChEMBL	ChEMBL (version 27) chemical database contains over 1.9 million specific compounds.	https://www.ebi.ac.uk/chembl/	Stokes et al., 2020
Drug target commons (DTC)	DTC crowdsourcing database contains 204,901 annotated bioactivity data points among 4,276 compounds and 1,007 specific protein targets.	https://drugtargetcommons.fimm.fi/	Beck et al., 2020
BindingDB	BindingDB database of measured binding affinities contains 2,061,017 binding data for 8,160 protein targets and 907,259 small molecules.	http://www.bindingdb.org/bind/index.jsp	Beck et al., 2020
DrugBank	DrugBank pharmaceutical database contains detailed molecular information about drugs, their mechanisms, interactions and targets.	https://go.drugbank.com/releases/latest	Choi et al., 2020; Zeng et al., 2020
PDBbind	PDBbind database provides binding data of 21,382 biomolecular complexes, including protein-ligand (17,679), nucleic acid-ligand (136), protein-nucleic acid (973), and protein-protein complexes (2,594).	http://www.pdbbind.org.cn	Bai et al., 2020

The outbreak of severe acute respiratory syndrome coronavirus 2 (SARS-CoV-2) causing coronavirus disease (COVID-19) has been declared a global pandemic. By September 2021, more than 220 million people have been infected with SARS-CoV-2 and more than 4.5 million of those infected have died. In addition, several SARS-CoV-2 variants with mutations that increase their potential to contribute to the severity of the pandemic have emerged and are spreading around the globe (Zhang Y. et al., 2020).

Besides non-structural proteins, SARS-CoV-2 genome encodes four structural proteins: envelope (E), membrane (M), nucleocapsid (N), and spike (S) (Zhang Y. et al., 2020). S protein mediates entry of SARS-CoV-2 into the host cells by binding and fusing with the host’s cellular receptor, the angiotensin-converting enzyme 2 (ACE2). Mutations in S protein, particularly in its receptor binding domain (RBD) were shown to play a role in the increased transmissibility and infectivity of the emerging SARS-CoV-2 variants (Zahradnik et al., 2021).

Although several SARS-CoV-2 vaccines have been developed over the last few months, there are not many efficient and reliable drugs available for the treatment of SARS-CoV-2 infections. This is caused partially by the fact that the traditional drug discovery process may be time-consuming and costly to keep pace with the rapid spread of SARS-CoV-2 and its variants with increased transmissibility and other enhanced features.

Deep learning has been previously applied for the identification of a number of antiviral compounds, including antiviral peptides (Timmons and Hewage, 2021) and small drug-like compounds with the potential to inhibit HIV-1 (Andrianov et al., 2021).

The computational approaches employing deep learning will aid also faster discovery of novel and active potential inhibition agents against SARS-CoV-2 and its emerging variants.

High-throughput technologies have generated an increasing amount of data in chemoinformatics. As a result, it is believed that the application of the recent deep learning advances into the drug discovery process will lead to novel, more reliable and efficient therapeutics against SARS-CoV-2.

## Antimicrobial Drugs Identified by Deep Learning

Deep learning can reduce time and costs of the drug discovery process, particularly in its early stages. Consequently, deep learning-based approaches have been successfully used to identify novel antimicrobial compounds against a wide variety of pathogenic microorganisms, including bacteria, protozoan parasites and viruses.

Training of the deep learning model to identify molecules active against antibiotic-resistant bacteria led to the discovery of Halicin and eight additional potential antibiotics from the ZINC database (Table 1; Stokes et al., 2020). Interestingly, these compounds identified by deep learning are all structurally divergent from conventional antibiotics (Stokes et al., 2020). Subsequent tests revealed strong antibacterial activity of Halicin against a number of antibiotic-resistant bacteria, including Carbapenemase-producing Enterobacterales, *Mycobacterium tuberculosis*, *Acinetobacter baumannii*, and *Clostridioides difficile* (Stokes et al., 2020).

In parasite research, deep learning has been applied to predict new antimalarial drug candidates. Neves et al. (2020) employed deep learning to obtain binary, continuous Quantitative Structure-Activity Relationships (QSAR) models using datasets extracted from ChEMBL database (Table 1). QSAR mathematical models can predict the relationship between the structure of a molecule and biological activity or physicochemical property. This study led to the discovery of two new families of the potential next generation antimalarial drugs with activity against *Plasmodium* causing malaria at nanomolar concentrations and low cytotoxicity in mammalian cells (Neves et al., 2020).

Deep learning has been also applied for the identification of a number of antiviral compounds. Timmons and Hewage developed a novel method called ENNAVIA, which employs deep learning and chemoinformatics, to identify peptides with low toxicity and excellent biological activity. The peptides identified in this study represent promising candidates for antiviral drugs (Timmons and Hewage, 2021). Furthermore, deep learning in combination with molecular modeling has been applied for the identification of three small drug-like compounds from millions of molecules in the ZINC15 database (Andrianov et al., 2021). Based on machine learning, molecular docking, molecular dynamics and quantum chemical calculations, the compounds identified in this study are promising HIV-1 drugs with the potential to block CD4-binding site of the viral envelope protein, thus inhibiting HIV-1 entry (Andrianov et al., 2021). Li et al. have developed a dual-channel deep neural network for identifying variable-length antiviral peptides (DeepAVP) which could block entry of the virus into the host cell (Li et al., 2020). Deep learning has been also used for the prediction of plant-exclusive virus-derived small interfering RNAs (PVsiRNAPred) (He et al., 2019).

## Deep Learning in Tackling Severe Acute Respiratory Syndrome Coronavirus 2

Reliable and efficient computing methods employing deep learning are urgently needed for the discovery of drugs against SARS-CoV-2 and its emerging variants.

Drug repurposing is considered to be among the fastest and most promising methods for identification of effective SARS-CoV-2 treatments. A good example of the drug repurposing involving deep learning is a recent work by Zhang Y. et al. (2020). This study employed a deep learning-based drug-target interaction model called Molecule Transformer-Drug Target Interaction (MT-DTI) utilizing chemical sequences [simplified molecular-input line-entry system (SMILES) strings] and amino acid (AA) sequences as the input (Figure 1). MT-DTI model was trained with a combined and curated chemical-protein pairs from BindingDB and Drug Target Commons (DTC) databases (Table 1). This study led to identification of several commercially available antiviral drugs with the potential to interact also with the SARS-CoV-2 proteins (Beck et al., 2020). Subsequent experiments showed that several of the antiviral agents identified by MT-DTI model could be potentially used to treat SARS-CoV-2 (Beck et al., 2020). These include Atazanavir (Kd 94.94 nM), Remdesivir (Kd 113.13 nM), and Kaletra (Lopinavir/Ritonavir) (Table 2). Atazanavir, showing an inhibitory potency against SARS-CoV-2 3-C like proteinase is an antiviral drug used for the treatment of the human immunodeficiency virus (HIV) infections. Remdesivir has been previously predicted to act against SARS-CoV-2. Furthermore, Lopinavir and Ritonavir were shown to target viral proteinases (Beck et al., 2020). The BindingDB is a public database containing measured binding affinities for three types of coronaviruses, SARS-CoV-2, SARS-CoV and MERS-CoV.^1^

**TABLE 2:** Drug candidates against SARS-CoV-2.

**Drug**	**Molecular formula**	**Structural formula**	**SMILES**	**Target**	**References**
Atazanavir	C_3__8_H_5__2_N_6_O_7_	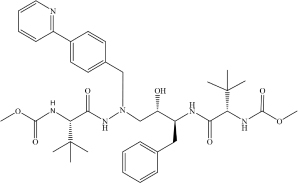	COC(N[C@@H](C(C)(C)C)C(NN (C[C@H](O)[C@H](CC1 = CC = CC = C1)NC([C@H](C(C)(C)C)NC (OC) = O) = O)CC(C = C2) = CC = C2C3 = NC = CC = C3) = O) = O	3C-like proteinase	Beck et al., 2020
Remdesivir	C_2__7_H_3__5_N_6_O_8_P	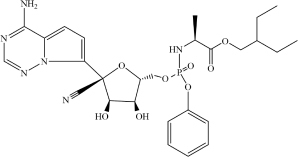	CP = O.COC1 = CC = CC = C1.O = C(OCC(CC)CC)[C@H](C)NC.NC2 = NC = NN3C2 = CC = C3[C@@]4 (C#N)[C@H](C)[C@H](C)[C@@H] (C[O])O4.[OH].[OH]	3C-like proteinase	Beck et al., 2020
Kaletra (Lopinavir/Ritonavir)	C_7__4_H_9__6_N_1__0_O_1__0_S_2_ (C_3__7_H_4__8_N_4_O_5_/C_3__7_ H_4__8_N_6_O_5_S_2_)	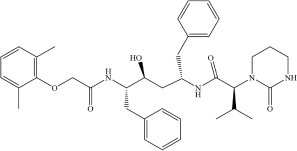	CC1 = CC = CC(C) = C1OCC(N[C@@H](CC2 = CC = CC = C2)[C@@H](O)C[C@H](CC3 = CC = CC = C3)NC([C@H](C(C)C) N4C(NCCC4) = O) = O) = O	Helicase	Beck et al., 2020
		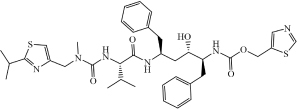	CC(C)C1 = NC(CN(C)C(N[C@@H] (C(C)C)C(N[C@@H](CC2 = CC = CC = C2)C[C@H](O)[C@H](CC3 = CC = CC = C3)NC(OCC4 = CN = CS4) = O) = O) = O) = CS1		
Enalaprilat	C_1__8_H_2__4_N_2_O_5_	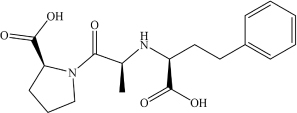	OC([C@H]1N(C([C@H](C)N[C@H](C(O) = O)CCC2 = CC = CC = C2) = O)CCC1) = O	ACE2	Choi et al., 2020
Venetoclax	C*45*H*50*ClN*7*O*7*S	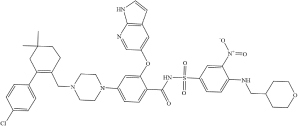	ClC1 = CC = C(C2 = C(CN3CC N(C4 = CC = C(C(NS(C5 = CC = C(NCC6CCOCC6)C([N + ]([O-]) = O) = C5)(= O) = O) = O)C(OC 7 = CC(C = CN8) = C8N = C7) = C4)CC3)CCC(C)(C)C2)C = C1	TMPRSS2 ACE2	Choi et al., 2020
Posaconazole	C_3__7_H_4__2_F_2_N_8_O_4_	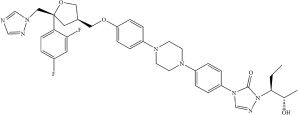	FC1 = CC(F) = CC = C1[C@@]2 (CN3N = CN = C3)C[C@H](COC 4 = CC = C(N5CCN(C6 = CC = C(N 7C = NN([C@@H](CC)[C@@H](O) C)C7 = O)C = C6)CC5)C = C4)CO2	TMPRSS2 ACE2	Choi et al., 2020
Daclatasvir	C_4__0_H_5__0_N_8_O_6_	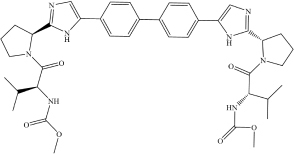	COC(N[C@@H](C(C)C)C(N1[C@H] (C2 = NC = C(C3 = CC = C(C4 = C C = C(C5 = CN = C([C@H]6N(C([C@ @H](NC(OC) = O)C(C)C) = O)CCC6)N 5)C = C4)C = C3)N2)CCC1) = O) = O	TMPRSS2 ACE2	Choi et al., 2020
Ombitasvir	C_5__0_H_6__7_N_7_O_8_	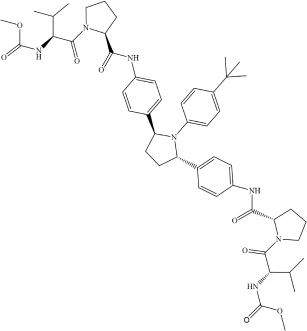	CC(C)(C)C(C = C1) = CC = C1N([C@ H](C2 = CC = C(NC([C@@H]3CCCN 3C([C@H](C(C)C)NC(OC) = O) = O) = O)C = C2)CC4)[C@@H]4C5 = CC = C(NC([C@H]6N(C([C@@H](NC(O C) = O)C(C)C) = O)CCC6) = O)C = C5	TMPRSS2 ACE2	Choi et al., 2020
Toremifene	C_2__6_H_2__8_ClNO	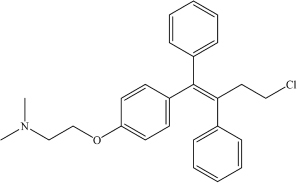	CN(C)CCOC1 = CC = C(/C(C2 = CC = CC = C2) = C(C3 = C C = CC = C3)/CCCl)C = C1	–	Zeng et al., 2020
Niclosamide	C_1__3_H_8_Cl_2_N_2_O_4_	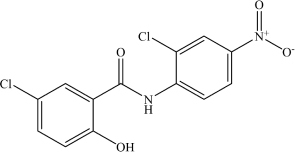	ClC1 = CC = C(O)C(C(NC2 = CC = C([N + ]([O-]) = O)C = C2Cl) = O) = C1	–	Zeng et al., 2020
Dexamethasone	C_2__2_H_2__9_FO_5_	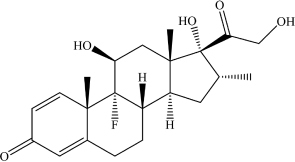	O = C1C = C[C@@]2(C)C(CC[C@] ([C@@](C[C@@H](C)[C@]3(O)C(C O) = O)([H])[C@]3(C)C[C@@H]4O) ([H])[C@@]24F) = C1	–	Zeng et al., 2020
Indomethacin	C_1__9_H_1__6_ClNO_4_	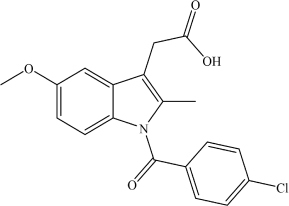	COC1 = CC = C(N(C(C2 = CC = C(Cl)C = C2) = O)C(C) = C3 CC(O) = O)C3 = C1	–	Zeng et al., 2020
Pralatrexate	C_2__3_H_2__3_N_7_O_5_	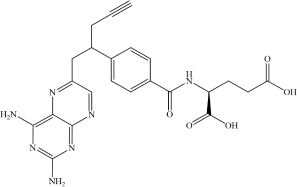	NC1 = C2C(N = CC(CC(C3 = CC = C(C(N[C@H](C(O) = O) CCC(O) = O) = O)C = C3) CC#C) = N2) = NC(N) = N1	RdRp	Zhang H. et al., 2020
Azithromycin	C_3__8_H_7__2_N_2_O_12_	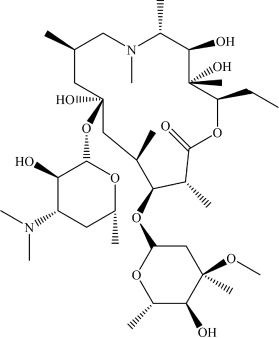	CN([C@H](C)[C@@H](O)C(C)(O)[C @@H](CC)O1)C[C@H](C)C[C@@](O[C@H]2[C@H](O)[C@@H](N(C)C)C[C@@H](C)O2)(O)C[C@@H](C)[C @H](O[C@H]3O[C@@H](C)[C@H](O)[C@](C)(OC)C3)[C@@H](C)C1 = O	RdRp	Zhang H. et al., 2020
Palmatine	C_2__1_H_2__2_NO_4_^+^	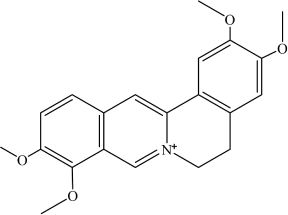	COC1 = C(OC)C(C = [N + ] (CCC2 = C3C = C(OC)C(OC) = C2)C3 = C4) = C4C = C1	Mpro enzyme of SARS-CoV-2	Joshi et al., 2020
Sauchinone	C_2__0_H_2__0_O_6_	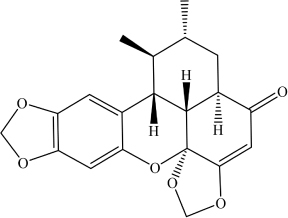	O = C1C = C2[C@]3(OCO2)[C@ @]4([H])[C@@]1([H])C[C@@H](C)[C@H](C)[C@@]4([H])C5 = CC (OCO6) = C6C = C5O3	Mpro enzyme of SARS-CoV-2	Joshi et al., 2020

*Table shows molecular and structural formulas, simplified molecular-input line-entry system (SMILES) strings and corresponding targets of the potential drugs against SARS−CoV−2.*

MT-DTI model was also used to select compounds from 1,400 approved drugs in DrugBank and ZINC databases (Table 1) with strong affinity to the host cell targets crucial for viral infection (Zahradnik et al., 2021). This approach led to identification of drugs candidates with a strong binding affinity (Kd < 100 nM) against ACE2 receptor and transmembrane protease serine 2 (TMPRSS2) (Zahradnik et al., 2021). Drug candidates identified in this study include an ACE2 inhibitor Enalaprilat (Kd 1.46 nM) and several drugs with predicted strong affinity for TMPRSS2, namely Venetoclax (Kd 6.12 nM), Posaconazole (Kd 17.11 nM), Daclatasvir (Kd 6.65 nM), and Ombitasvir (Kd 5.91 nM) (Table 2). Strong affinity of Enalaprilat for ACE2 suggests that it might prevent the entry of SARS-CoV-2 to human cells. Notably, two of the drug candidates identified, namely Daclatasvir and Ombitasvir, are known Hepatitis C virus (HCV) inhibitors, thus suggesting that they may act against both HCV and SARS-CoV-2 (Zahradnik et al., 2021). The DrugBank has collected data for 65 drugs against 385 drug targets, which is web accessible at https://go.drugbank.com/covid-19.

Zeng et al. used deep learning-based knowledge graph to select promising SARS−CoV−2 drug candidates (Zeng et al., 2020). Knowledge graph in this study encompasses 15 million edges across 39 types of relationships connecting expression patterns, genes, pathways, drugs and diseases and incorporates data from over 20 million PubMed articles and the DrugBank database (Table 1). Deep learning employed to learn the representation of nodes and relationships in this knowledge graph led to identification of 41 promising drug candidates, including Toremifene, Niclosamide, Dexamethasone and Indomethacin (Table 2; Beck et al., 2020). Toremifene is a selective estrogen receptor modulator, which has shown antiviral activity against a number of viruses, including SARS-CoV-2. Dexamethasone is an anti-inflammatory agent with the potential to treat SARS-CoV-2 infections (Beck et al., 2020). Niclosamide, a drug used to treat tapeworm and an anti-inflammatory drug Indomethacin were also shown to have antiviral activity *in vitro*. The 41 promising drug candidates identified in this study (including Toremifene, Niclosamide, Dexamethasone and Indomethacin) were also validated by gene expression and proteomics of cells infected with SARS-CoV-2 (Beck et al., 2020).

A hybrid deep learning and molecular simulation-based screening procedure was used to select drug candidates targeting RNA-dependent RNA polymerase (RdRp) from 1906 approved drugs, recently (Choi et al., 2020). Commercially available drug candidates, Pralatrexate and Azithromycin, (Table 2) identified in this study were confirmed to inhibit SARS-CoV-2 replication *in vitro* (Choi et al., 2020). While Pralatrexate was shown to act after entry of the virus into the cells, Azithromycin was active at both the entry and post-entry of SARS-CoV-2 into the host cells (Choi et al., 2020).

Bai et al. developed MolAICal software tool combining deep learning model and classical algorithm for identification of drugs interacting with 3D pocket of protein targets (Bai et al., 2020). Deep learning model of MolAICal software was trained using approved drug fragments in PDBbind database and drug-like molecules in the ZINC database (Table 1). Drug design functions of MolAICal software were demonstrated using the membrane protein glucagon receptor (GCGR) and SARS−CoV−2 main protease (Mpro) (Zeng et al., 2020).

Ton et al. (2020) developed a Deep Docking (DD) deep learning platform which uses QSAR models for screening of potential drug candidates in the ZINC database (Table 1). This approach led to the identification of 1,000 potential ligands for SARS−CoV−2 Mpro (Ton et al., 2020).

Deep learning and molecular docking methods were developed for screening of natural compounds against SARS-CoV-2 Mpro in the ChEMBL database (Table 1; Bai et al., 2020). ChEMBL database is an open large-scale chemical database of bioactive molecules, containing 8,200 potential anti-SARS-CoV-2 drug candidates. This study led to the identification of two natural compounds with potential as therapeutics against SARS-CoV-2, namely Palmatine (Kd 1096.4 nM) and Sauchinone (Table 2) (Kd 389.05 nM). Palmatine and Sauchinone are an alkaloid and a lignan, respectively, with previously shown pharmacological properties. Furthermore, both Palmatine and Sauchinone form a stable complex with SARS-CoV-2 Mpro and have been predicted to inhibit SARS-CoV-2 (Bai et al., 2020).

Deep learning combined with multiple sequence alignment drug-likeness screening, molecular docking, chemical space mapping and molecular dynamics simulation was also used to identify drug candidates by screening 1528 anti-HIV-1 compounds against 3-chymotrypsin-like cysteine protease (3CLpro) of SARS-CoV-2 (Nand et al., 2020).

Given the lack of therapeutics against SARS-CoV-2, deep learning approaches combined with other computational methods will play an important role in the identification of potential drugs targeting SARS-CoV-2. Compounds selected by deep learning will subsequently undergo standard clinical evaluation.

## Discussion

Deep learning has a number of advantages compared to more conventional methods, including its ability to learn complex features independently. Although deep learning has played an important role in the identification of novel drugs against a wide range of pathogens, including SARS-CoV-2, many challenges still remain.

The connection between the data fed into the deep learning model and the delivered output is inscrutable, which hidden inside is a so-called black box. Deep neural network due to its black-box nature therefore often lacks interpretability. Therefore, the interpretability of the future neural networks on the output results will be a key factor in understanding the logic of machine. This will aid analysis of the chemical compounds identified by deep learning and better design of the drug discovery studies.

Furthermore, the input data affects the prediction performance of the deep learning model. Consequently, a large, standardized and reliable biochemical dataset is necessary to achieve better learning of the deep learning model. Development of a large open dataset in the future will enable potential standardization of the deep learning-based drug discovery.

Antibody-based therapy represents an interesting SARS-CoV-2 treatment option. Deep learning models have been developed for the discovery and design of therapeutic antibodies (Mason et al., 2021; Saka et al., 2021). Thus, drug repositioning and screening from computational libraries containing a massively diverse antibody sequences could be used to engineer anti-viral SARS-CoV-2 treatment.

Furthermore, most recent studies describe methodologies separately and test them individually. Application of deep learning to combine chemoinformatics with other types of data, such as imaging, cellular and molecular biology data for integrative analysis would be an important direction for future research. To this end, it might be necessary to identify the best neural network architecture for handling those vast troves of data.

We believe that integrative and systematic analysis will be important for future deep learning-based drug discovery that involves complicated large biological, chemical and clinical datasets. Using such large datasets to streamline and accelerate drug discovery, deep learning will be crucial not only for the identification of drug candidates against SARS-CoV-2 but also against a broad spectrum of other emerging and reemerging pathogens.

## Author Contributions

All authors contribute to the writing and reviewing the manuscript.

## Conflict of Interest

The authors declare that the research was conducted in the absence of any commercial or financial relationships that could be construed as a potential conflict of interest.

## Publisher’s Note

All claims expressed in this article are solely those of the authors and do not necessarily represent those of their affiliated organizations, or those of the publisher, the editors and the reviewers. Any product that may be evaluated in this article, or claim that may be made by its manufacturer, is not guaranteed or endorsed by the publisher.

## Footnotes

1
https://www.bindingdb.org/bind/Covid19.jsp
